# A Semi-automatic Diagnosis of Hip Dysplasia on X-Ray Films

**DOI:** 10.3389/fmolb.2020.613878

**Published:** 2020-12-17

**Authors:** Guangyao Yang, Yaoxian Jiang, Tong Liu, Xudong Zhao, Xiaodan Chang, Zhaowen Qiu

**Affiliations:** ^1^Department of Computer Science and Technology, College of Information and Computer Engineering, Northeast Forestry University, Harbin, China; ^2^Department of Radiology, Affiliated Zhongshan Hosptial of Dalian University, Dalian, China; ^3^Heilongjiang Tuomeng Technology Co. Ltd., Harbin, China

**Keywords:** hip joint, dysplasia, x-ray, manual segmentation, automatic angle measurement, density descending clustering

## Abstract

**Background:** Diagnosis of hip joint plays an important role in early screening of hip diseases such as coxarthritis, heterotopic ossification, osteonecrosis of the femoral head, etc. Early detection of hip dysplasia on X-ray films may probably conduce to early treatment of patients, which can help to cure patients or relieve their pain as much as possible. There has been no method or tool for automatic diagnosis of hip dysplasia till now.

**Results:** A semi-automatic method for diagnosis of hip dysplasia is proposed. Considering the complexity of medical imaging, the contour of acetabulum, femoral head, and the upper side of thigh-bone are manually marked. Feature points are extracted according to marked contours. Traditional knowledge-driven diagnostic criteria is abandoned. Instead, a data-driven diagnostic model for hip dysplasia is presented. Angles including CE, sharp, and Tonnis angle which are commonly measured in clinical diagnosis, are automatically obtained. Samples, each of which consists of these three angle values, are used for clustering according to their densities in a descending order. A three-dimensional normal distribution derived from the cluster is built and regarded as the parametric model for diagnosis of hip dysplasia. Experiments on 143 X-ray films including 286 samples (i.e., 143 left and 143 right hip joints) demonstrate the effectiveness of our method. According to the method, a computer-aided diagnosis tool is developed for the convenience of clinicians, which can be downloaded at http://www.bio-nefu.com/HIPindex/. The data used to support the findings of this study are available from the corresponding authors upon request.

**Conclusions:** This data-driven method provides a more objective measurement of the angles. Besides, it provides a new criterion for diagnosis of hip dysplasia other than doctors' experience deriving from knowledge-driven clinical manual, which actually corresponds to very different way for clinical diagnosis of hip dysplasia.

## 1. Introduction

Hip is one of the largest joint in human body. Its normal structure maintains people's daily activities. Hip dysplasia, which is thought to be hereditary (Harsanyi et al., 2020), is the main cause of hip osteoarthritis (Ganz et al., 2008). If the surface of acetabular is too small or tilts for a long time, the femoral head cannot be completely covered. Therefore, it will lead to uneven pressure, which will develop into irreversible osteoarthritis in the end (Yasuda et al., 2020). Early screening of hip dysplasia for adults followed by proper clinical management can not only save medical resources but also keep patients away from the pain of operation (Gala et al., 2016).

Although three dimensional structure of hip joint can be derived from CT and MRI images, it has to be faced with relatively high fees of medical check and high radiation. Therefore, the radiograph of pelvis from a X-ray film becomes the main early diagnosis of hip dysplasia (Kayaalp et al., 2020; Powell et al., 2020). Inevitably, the ever-growing numbers of X-ray films increase the burden of radiologists. An automatic method or tool for them to make auxiliary measurement or even diagnosis is needed.

As to automatic measurement or diagnosis of hip dysplasia on X-ray films, there are three problems. Firstly, it is difficult to segment the hip automatically considering the inhomogeneous intensity derived from the image superimposition of acetabulum. Thus, most of the existing methods are aimed at femur segmentation. Xie et al. (2014) extracted shape features to segment the proximal femur. Wei et al. (2020) improved deep convolutional generative adversarial network (DCGAN) for segmentation of femur. Liu et al. (2020) proposed a Pyramid Nonlocal UNet (PN-UNet) for automatic misshapen landmark detection and neighboring patch segmentation. However, boundaries of acetabulum and femur were not clearly marked. Secondly, prevailing diagnosis of hip dysplasia mainly depends on the manual measurement of angles on X-ray films (Simone and Klaus, 2014). It is difficult to automatically calibrate feature angles including center-edge (CE) angle, sharp angle, Tonnis angle, etc., which are commonly measured in clinical diagnosis (Beltran et al., 2013). Thirdly, a diagnostic manual is consulted to test whether hip development is normal or not (Harper et al., 2020; Ömeroglu et al., 2020). Automatic and objective indicators for clinical diagnosis of hip dysplasia are needed.

**Figure 1:**
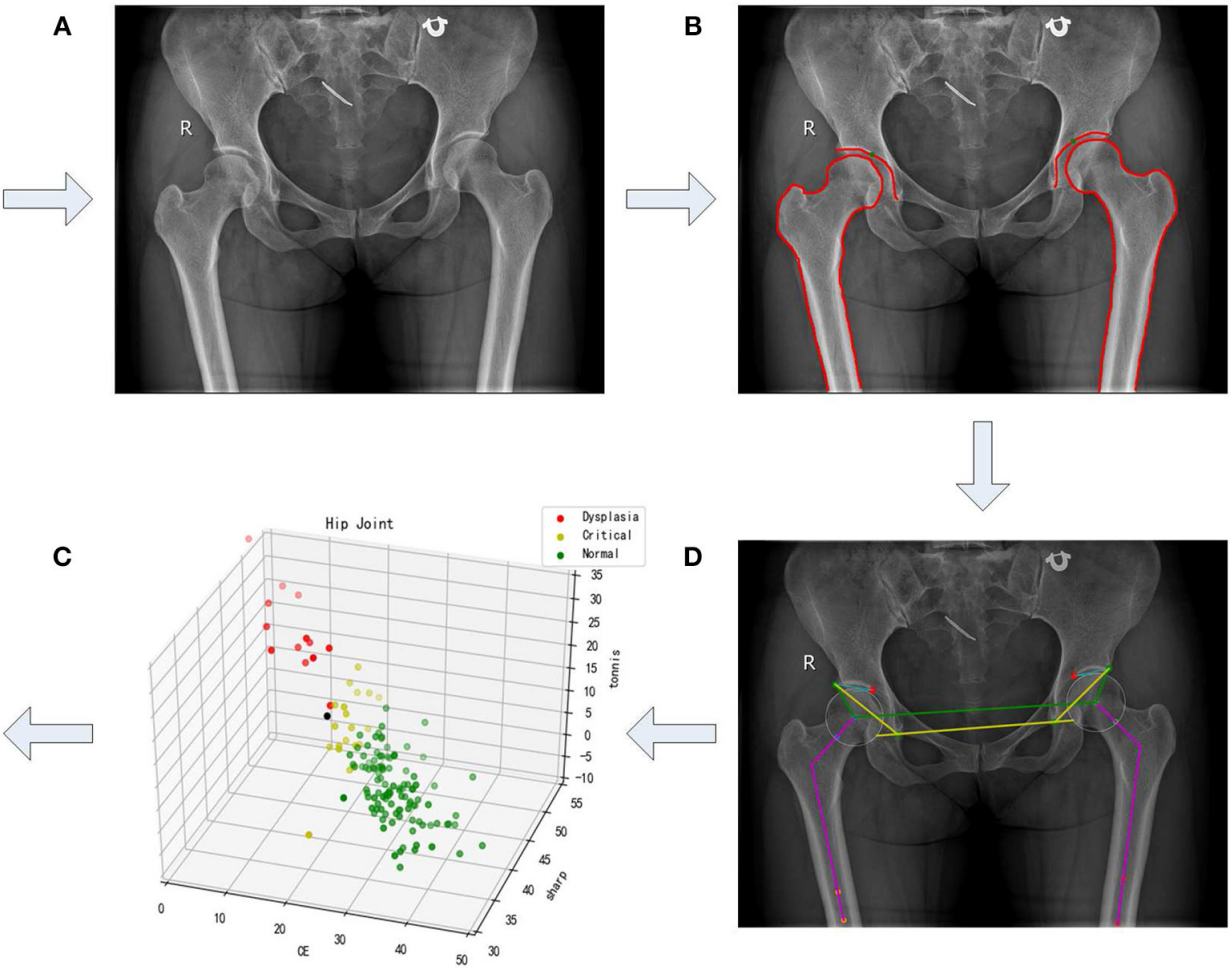
A framework of semi-automatic method for diagnosis of hip dysplasia on X-ray films. **(A–D)** correspond to four steps of the framework. **(A)** refers to an original X-ray file of hip joint. **(B)** represents the manually marked results. **(C)** corresponds to the automatic angle measurement. **(D)** shows the corresponding scatter in the three-dimensional space with its three directions representing CE, sharp, and Tonnis angle.

In this paper, we propose an approach for semi-automatic diagnosis of hip dysplasia on X-ray films. The corresponding processing framework is shown in Figure 1. Due to the difficulty of accurate acetabulum segmentation, contours of hip joint including acetabulum, femoral head, and the upper side of thigh-bone are manually delineated. Then, feature angles including CE, sharp, and Tonnis are automatically extracted from the marked contour. Thereafter, a scatter point is obtained in three-dimensional space according to these feature angles. These procedure is repeated using 286 samples representing either left or right hip joints. Finally, previously proposed clustering method using density in a descending order is presented on these scatter points, and a model representing normal hip development is made for diagnosis of hip dysplasia.

## 2. Method

The dataset representing either normal development of hip joint or hip dysplasia is provided, which contains 143 X-ray films including 286 samples from 143 left and 143 right hip joints. That is, an X-ray film is considered as two parts, each of which contains either imaging of left hip joint or the right one. Manual delineation of acetabulum, femoral head, and the upper side of thigh-bone is made on each film. Actually, we follow the framework presented in Figure 1 to establish the model representing normal hip development for diagnosis of hip dysplasia. More details can be seen in the following subsections.

### 2.1. Automatic Extraction of CE

**Figure 2:**
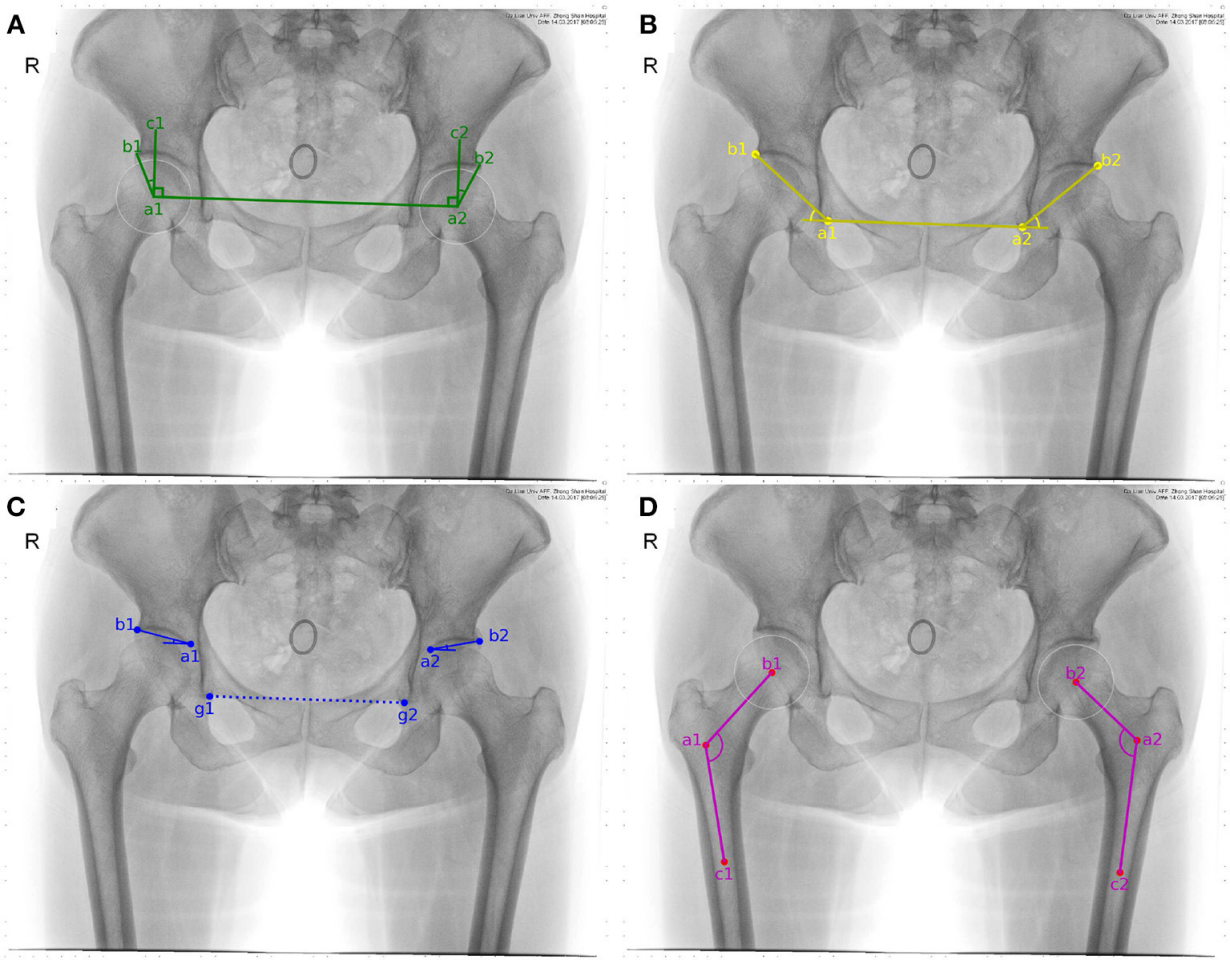
Sketch maps of feature angles. **(A–D)** correspond to CE, sharp, Tonnis, and caput collum diaphysis (CCD) angle, respectively. Considering that CCD only measures coxa valga or coxa vara on femoral head, it is discarded in the following study.

According to the result of manual delineation, some feature points can be obtained, which help to form feature angles automatically. Sketch maps of these feature angles are illustrated in Figure 2. CE angle is commonly considered to be the first feature angle for clinical diagnosis. As shown in Figure 2A, it refers to the angle between two lines. One line is derived from the connection between the central point of femoral head (i.e., *a*_1_ or *a*_2_) and the external upper edge of acetabulum (i.e., *b*_1_ or *b*_2_). The other line refers to the vertical of the line after connecting the two central points of right and left femoral head which two are labeled as *a*_1_ and *a*_2_, respectively.

**Figure 3:**
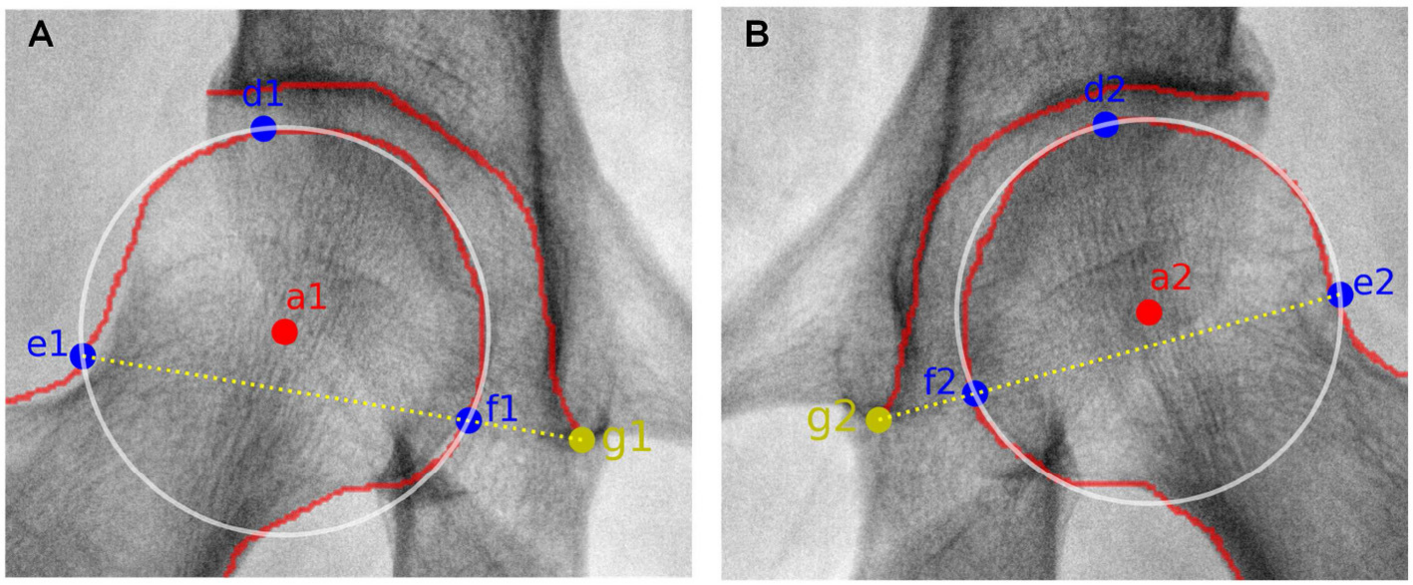
Sketch maps of getting the circle representing the femoral head. **(A,B)** correspond to results from the right and left femoral head. *a*_1,2_ refer to the central point of the right and left femoral head, respectively. *d*_1,2_ and *e*_1,2_ represent the uppermost and outermost points of the right and left femoral head, respectively. *g*_1,2_ correspond to the lower edge points of the right and left acetabulum, respectively. As to *f*_1,2_, they are the intersection points of the connection line (i.e., *e*_1,2_ and *g*_1,2_) and the contour of right and left femoral head, respectively.

In order to automatically obtain CE angle, *a*_1_, *a*_2_, *b*_1_, and *b*_2_ have to be determined in advance. According to manual delineation, the external upper edge of acetabulum, i.e., *b*_1_ and *b*_2_, can be apparently labeled. As to the central point of the femoral head, it is regarded as the center of a circle which can cover the femoral head. Considering that three points which are not collinear can determine a circle in a plane, three feature points have to be automatically labeled. Here, the uppermost and outermost point on the contour of the femoral head are selected, as labeled with *d*_1_, *d*_2_, *e*_1_, and *e*_2_ shown in Figure 3. Taking the inflection points of femoral head and femoral neck as the boundary, *e*_1_ and *e*_2_ can be simply found. The third feature point can be obtained by connecting *e*_1,2_ and *g*_1,2_, which refer to the lower edge point of right and left acetabulum. That is the intersection of the connection line and the contour of femoral head denoted as *f*_1_ and *f*_2_ in Figure 3. Thus, the central point of the femoral head are obtained and labeled as *a*_1_ and *a*_2_ in Figure 3. Correspondingly, CE angle can be expressed as

(1) $$\begin{array}{l} \theta_{CE}=\arccos\frac{\vec{a_{1,2}c_{1,2}}\cdot \vec{a_{1,2}b_{1,2}}}{\left|\vec{a_{1,2}c_{1,2}}|\;|\vec{a_{1,2}b_{1,2}}\right|}. \end{array}$$

### 2.2. Automatic Extraction of Sharp

Sharp angle is also regarded as a feature angle for clinical diagnosis. As shown in Figure 2B, it also refers to the angle between two lines. One line is derived from the connection between the lower edge point of the right acetabulum (i.e., *a*_1_) and that of the left acetabulum (i.e., *a*_2_). The other line refers to the connection between the lower edge point of the acetabulum (i.e., *a*_1_ or *a*_2_) and the external upper edge of acetabulum (i.e., *b*_1_ or *b*_2_). Correspondingly, sharp angle is expressed as follows. That is,

(2) $$\begin{array}{l} \theta_{sharp}=\arccos\frac{\vec{a_{1,2}b_{1,2}}\cdot \vec{a_{2,1}a_{1,2}}}{\left|\vec{a_{1,2}b_{1,2}}|\;|\vec{a_{2,1}a_{1,2}}\right|}. \end{array}$$

### 2.3. Automatic Extraction of Tonnis

Tonnis angle is also considered to be a feature angle for clinical diagnosis of hip dysplasia. As illustrated in Figure 2C, it also refers to the angle contained by two lines. One line is derived from the connection between the external upper edge of acetabulum (i.e., *b*_1_ or *b*_2_) and the lower edge of the weight-bearing area of acetabulum (i.e., *a*_1_ or *a*_2_). The other line refers to the connection between the lower edge point of the right acetabulum (i.e., *g*_1_) and that of the left acetabulum (i.e., *g*_2_).

In order to automatically obtain Tonnis angle, *a*_1_ and *a*_2_ have to be pointed out in advance. In fact, the lower edge of the weight-bearing area of acetabulum can be easily found in an X-ray film containing hip joint, for the weight-bearing area of acetabulum keeps an obvious contrast to its surrounding area (see Figure 1A). Therefore, these two points can be labeled during manual delineation (see green points in Figure 1B). Correspondingly, Tonnis angle is expressed as

(3) $$\begin{array}{l} \theta_{Tonnis}=\arccos\frac{\vec{a_{1,2}b_{1,2}}\cdot \vec{g_{2,1}g_{1,2}}}{\left|\vec{a_{1,2}b_{1,2}}|\;|\vec{g_{2,1}g_{1,2}}\right|}. \end{array}$$

### 2.4. Clustering Using Sample Density in a Descending Order

As to each sample derived from an X-ray film, its CE, sharp, and Tonnis angle can be automatically calculated using Equations (1), (2), and (3). Correspondingly, a three-dimensional scatter point can be obtained associated with this sample. This procedure combining manual delineation of contours with automatic extraction of angles is repeated *n* times, where *n* represents sample size. Thus, we get a three-dimensional scatter plot as shown in Figure 1D. Using previously proposed clustering method (Liu et al., 2019), the cluster corresponding to normal hip development is obtained. If samples within the cluster are considered to obey three-dimensional normal distribution, then a statistical model can be established. The corresponding probability density function is expressed as

(4) $$\begin{array}{l} p\left(x\right)=\frac{1}{{\left(2\pi \right)}^{\left(3/2\right)}|\Sigma {|}^{1/2}}e^{-\frac{1}{2}{\left(x-\mu \right)}^{T}\Sigma^{-1}\left(x-\mu \right)}, \end{array}$$

where *x* represents the vector ${\left(\theta_{CE},\theta_{sharp},\theta_{Tonnis}\right)}^{T}$. μ and Σ denote sample mean and covariance matrix, respectively.

## 3. Results

Experiments were conducted on 286 samples derived from 143 X-ray films of hip joint which contained 143 left and 143 right hip joint. The data was digital bilateral hip x-rays retrospectively collected from the Affiliated Zhongshan Hospital of Dalian University from January 2017 to January 2018. This study was approved by the hospital's ethics committee. All patient information was de-identified before data analysis.

**Figure 4:**
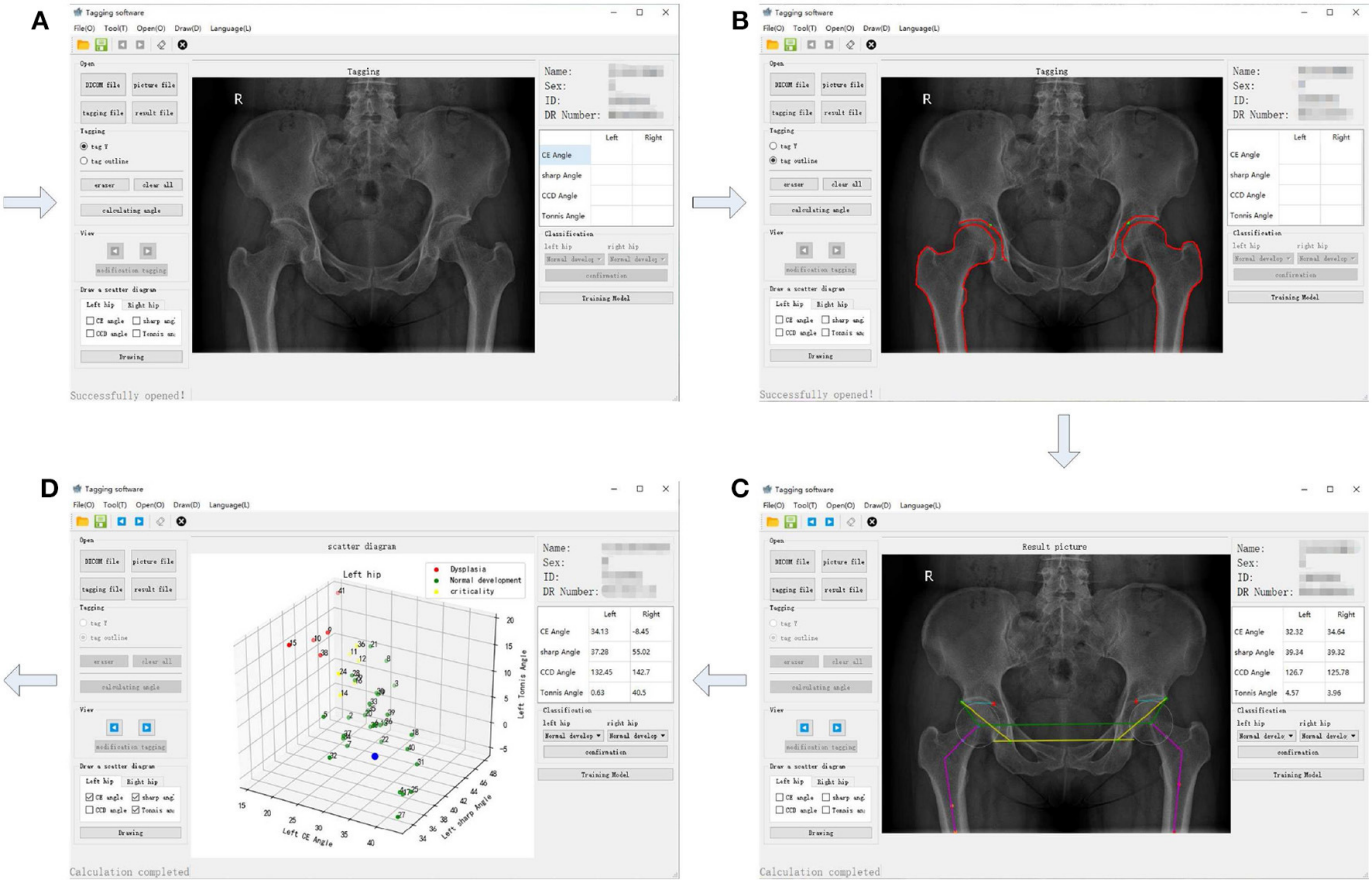
The graphical user interface (GUI) of the semi-automatic method for diagnosis of hip dysplasia on X-ray films. **(A–D)** correspond to four steps of the GUI.

The procedure shown in Figure 1 was accomplished using our own developed graphical user interface (GUI) listed in Figure 4. An X-ray film can be imported, as shown in Figure 4A. In Figure 4B, contours of acetabulum, femoral head, and the upper side of thigh-bone can be manually marked, together with the lower edge of the weight-bearing area of acetabulum. Then, feature angles including CE, sharp and Tonnis are automatically extracted, as illustrated in Figure 4C. Thereafter, a scatter point is projected into a three-dimensional space, which is composed of CE, sharp and Tonnis angle values calculated using Equations (1), (2), and (3), respectively. This procedure is repeated until enough scatter points have been got. Then, one can push the “Training Model" button shown in Figure 4D to establish the data-driven model for further clinical diagnosis of hip dysplasia.

**Figure 5:**
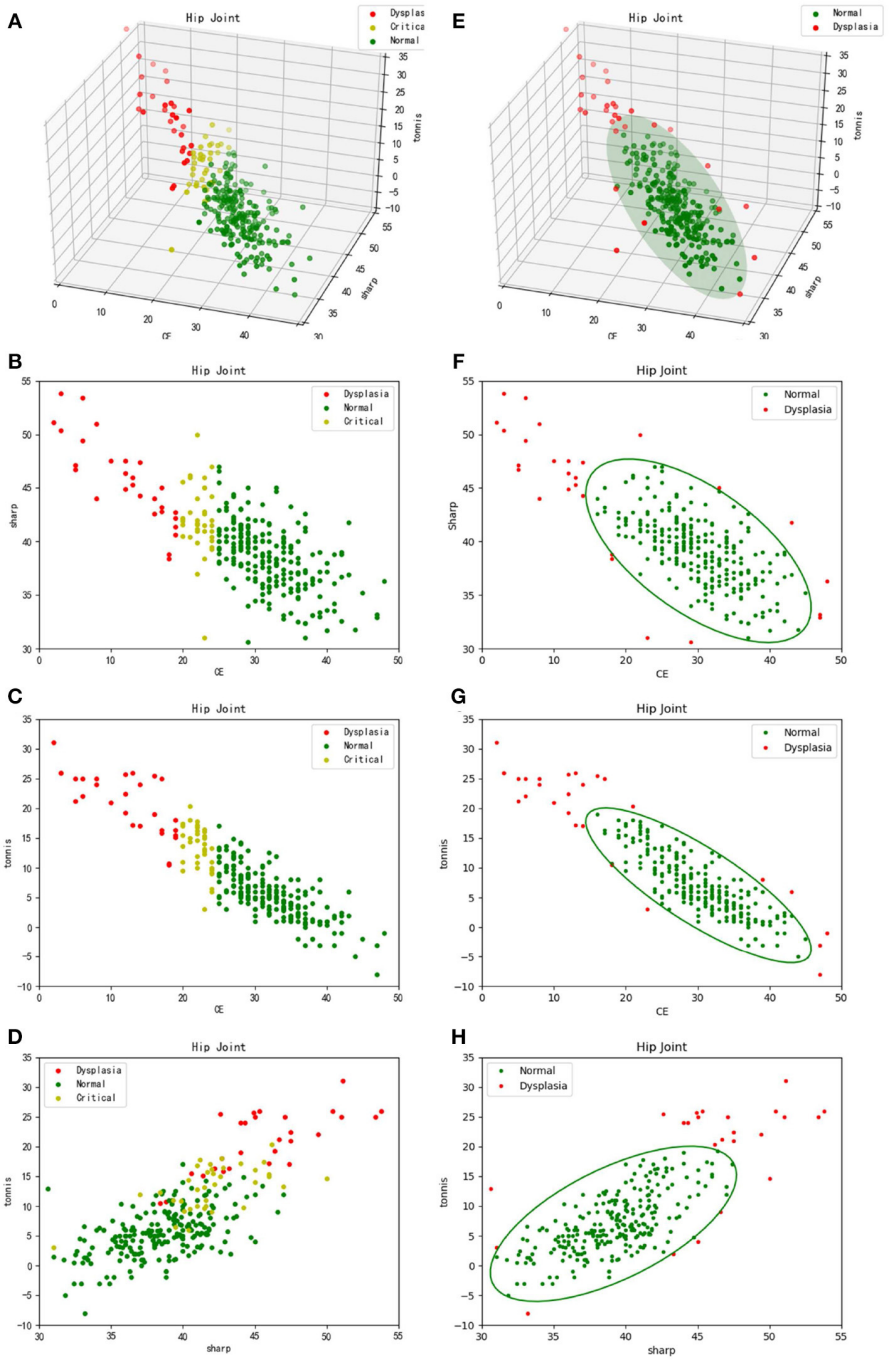
Three-dimensional scatter plots and their two-dimensional projection results. **(A–D)** refer to the traditional diagnosis of hip dysplasia deriving from knowledge-driven clinical manual in a three-dimensional space and its two-dimensional projection subspaces, respectively. Therefore, θ_*CE*_ is to be considered. That is, samples labeled with different colors are derived from different θ_*CE*_ scopes. **(E–H)** correspond to the new data-driven criterion for diagnosis of hip dysplasia in a three-dimensional space and its two-dimensional projection subspaces, respectively. The ellipsoid and corresponding projection ellipses refer to the parametric model.

Meanwhile, each CE, sharp, and Tonnis angle of the 286 samples were measured by a radiologist. The reference value of CE angle was considered as the knowledge-driven diagnostic criteria. Typically, a diagnosis of hip dysplasia was made, when $\theta_{CE}<20^{\circ }$. On the contrary, it was thought to be normal development of hip joint, when $\theta_{CE}>25^{\circ }$. Besides, it was considered as borderline dysplasia, when $20^{\circ }\leq \theta_{CE}\leq 25^{\circ }$. Therefore, 286 samples were labeled with color red, green and yellow, corresponding to abnormal, normal and borderline development of hip joint, respectively. Accordingly, the three-dimensional scatter plot and its two-dimensional projection results are listed in turn, as shown in Figures 5A–D. From these sub-figures, it can be seen that these samples obey normal distribution. However, plane or lines perpendicular to CE axis are considered to be the classification boundary using CE angle as the diagnostic criteria. Besides, it may be inappropriate even considering CE, sharp, and Tonnis angle at the same time, for the formed classification boundaries can be only perpendicular to coordinate axes. In fact, the appropriate classification boundary should be the plane perpendicular to the long axis of the ellipsoid derived from the three-dimensional normal distribution of samples.

Thus, previously proposed clustering method according to sample's density in a descending order (Liu et al., 2019) was utilized. In addition, samples within the cluster keeping the highest density peak, which represent normal development of hip joint, are considered to obey three-dimensional normal distribution. Using Equation (**4**), a model representing normal hip development is established for diagnosis of hip dysplasia, as shown in Figure 5E. Its two-dimensional projections are listed in turn, as shown in Figures 5F–H.

## 4. Discussions

We intend to make some simple discussions as follows. Firstly, it needs to be considered whether automatic segmentation can be made. In fact, we have utilized U-Net (Ronneberger et al., 2015) for discovering contours of acetabulum, femoral head, and the upper side of thigh-bone. Due to the limited sample size, the segmentation results were not good. Besides, it was found X-ray films from different digital radiography (DR) machines were quite different (see Figures 1, 2). That makes automatic segmentation more hard.

Secondly, it needs to be discussed whether the knowledge-driven diagnostic criteria is effective or not. CE, sharp and Tonnis angle are commonly used as the measurements of hip joint development. The typical thresholds for diagnosis of dysplasia are $\theta_{CE}<20^{\circ }$ and $\theta_{Tonnis}>10^{\circ }$ (Kosuge et al., 2013), which empirically represent the over-shallow acetabular and over-upward inclination of the weight-bearing area of acetabulum, respectively. In contrast, we proposed a data-driven diagnostic model for hip dysplasia, which took full account of sample distribution. After comparing the experimental results shown in Figure 5, it can be concluded that the data-driven criterion for diagnosis of hip dysplasia is more suitable, because it fits the sample distribution better.

Thirdly, outliers shown in Figure 5E need to be further considered. For those with small θ_*CE*_s but high θ_*sharp*_s and θ_*Tonnis*_s, diagnosis of hip dysplasia can be made. However, other outliers need to be further discussed. The reason why they are different from the traditional samples with hip dysplasia and whether they belong to new subtypes of hip dysplasia or not needs to be explained. Therefore, these cases should be carefully selected. Except for X-ray films, other imaging diagnosis and clinical diagnosis should be provided to test whether these special outliers belong to hip dysplasia or not.

## 5. Conclusion

Diagnosis of hip dysplasia plays a vital role in early screening of hip diseases. In this study, we proposed a semi-automatic method for diagnosis of hip dysplasia on X-ray films. Due to the complex appearances of hip joint imaging on X-ray films, a manual delineation was made on contours of acetabulum, femoral head and the upper side of thigh-bone. Furthermore, feature points were automatically or semi-automatically extracted. Then, feature angles were automatically obtained. Samples derived from three feature angles (i.e., CE, sharp and Tonnis) were used to accomplish clustering, which helped to establish a criterion model based on three-dimensional normal distribution for diagnosis of hip dysplasia. Besides, a GUI was provided for the convenience of clinicians. In future work, automatic segmentation of hip joint will be considered.

## Data Availability Statement

The raw data supporting the conclusions of this article will be made available by the authors, without undue reservation.

## Ethics Statement

The studies involving human participants were reviewed and approved by the ethics committee of the hospital. The patients/participants provided their written informed consent to participate in this study.

## Author Contributions

XZ, XC, and ZQ conceived the general project and supervised it. XZ initiated the idea, conceived the whole process, and finalized the paper. GY and TL were the principal developers. YJ collected the cases and made the manual delineation. All authors read and approved the final manuscript. All authors contributed to the article and approved the submitted version.

## Conflict of Interest

ZQ was employed by the company Heilongjiang Tuomeng Technology Co. Ltd., Harbin, China. The remaining authors declare that the research was conducted in the absence of any commercial or financial relationships that could be construed as a potential conflict of interest.
